# Navigating sign language learning: insights from hearing parents of deaf and hard-of-hearing children

**DOI:** 10.1093/jdsade/enaf059

**Published:** 2025-10-29

**Authors:** Jos Ritmeester, Beyza Sümer, Marije Boonstra, Maartje de Meulder, Belinda van der Aa, Floris Roelofsen

**Affiliations:** Institute for Logic, Language and Computation, University of Amsterdam, Amsterdam, The Netherlands; Department of Linguistics, Amsterdam Center for Language and Communication, University of Amsterdam, Amsterdam, The Netherlands; Research Department, Royal Auris Group, Rotterdam, The Netherlands; Research Group Speech and Language Therapy: Participation through Communication, Faculty of Healthy and Sustainable Living, HU University of Applied Sciences, Utrecht, The Netherlands; Research Department, Royal Auris Group, Rotterdam, The Netherlands; Institute for Logic, Language and Computation, University of Amsterdam, Amsterdam, The Netherlands

## Abstract

The importance of hearing parents of deaf and hard-of-hearing (DHH) children learning sign language is well documented. However, parents face many challenges in this learning process. This study investigates the experiences of Dutch hearing parents learning Dutch Sign Language (NGT) or Sign-supported Dutch through semi-structured interviews with 21 parents and 6 NGT teachers. The interviews explored parents’ and teachers’ perspectives on parental sign language courses, additional learning materials, and the challenges parents face in learning sign language. The findings highlight the value of DHH teachers and home-based initial courses, as well as the importance of courses aligning with the child’s developmental stage and extending beyond vocabulary level. Both parents and teachers appreciated learning materials that could be used together by parent and child but expressed a need for additional and more elaborate resources. Common challenges included language-specific difficulties, such as mastering sign order and adapting to a visual language, and external barriers, such as difficulties accessing courses and conflicting expert advice regarding the use of sign language. These findings underscore the need for more accessible courses, longer-duration support, and greater consistency among professionals in their advice. This would better support hearing parents in effectively learning sign language and ensuring their DHH children have full access to language from an early age.

Deaf and hard-of-hearing (DHH) children who receive early sign language input follow similar language milestones as hearing children at a comparable rate (Baker et al., 2016; Lillo-Martin & Henner, 2021; Mayberry & Squires, 2006). However, 90%–95% of DHH children are born to hearing parents (Mitchell & Karchmer, 2004), who are generally not sign language users. DHH children therefore often experience delays in receiving accessible language input, which can lead to poor language outcomes in several domains such as lexicon, phonology, and syntax that persist into adulthood (Cheng et al., 2023; Henner et al., 2016; Mayberry, 2007). Delayed accessible language exposure has also been found to correlate with negative changes in adult brain structures related to language learning, indicating that without early exposure, the brain’s language system fails to fully develop (Cheng et al., 2023). Besides affecting language development, delayed sign language input can negatively affect a DHH child’s cognitive and social–emotional development (Hall, 2017; Marschark & Hauser, 2012; Snoddon, 2015; Zarchy & Geer, 2023).

Creating an accessible language environment is essential for hearing parents of DHH children, and learning sign language as early as possible plays a critical role in this process. Research on both American Sign Language (ASL) and Dutch Sign Language (*Nederlandse Gebarentaal*, NGT) has shown that hearing parents who commit to learning sign language can achieve (high) intermediate proficiency (level B1/B2 of the Common European Framework of Reference for Languages). At this level, they are considered independent language users, capable of effectively communicating in sign language with their DHH child and DHH people in their child’s life (Oyserman & de Geus, 2021; Pontecorvo et al., 2024). These language skills are not only valuable for facilitating direct communication but also have a measurable impact on their child’s language development. Studies on ASL have demonstrated that parents’ sign proficiency positively correlates with their DHH child’s lexical, phonological, and syntactic development (Berger et al., 2023; Caselli et al., 2021; Chen Pichler et al., 2024). Furthermore, DHH children of hearing parents can achieve age-appropriate ASL vocabulary sizes if they are exposed to sign language by 6 months of age (Caselli et al., 2021). These findings underscore that, even as second-language learners, hearing parents can provide meaningful early sign language input for their DHH children.

This paper explores the experiences of hearing parents of DHH children learning sign language. Specifically, we aim to broaden the existing body of research, which primarily centers on ASL, by examining the experiences of Dutch parents learning NGT. The next subsection reviews previous work on hearing parents’ experiences with sign language learning, including the challenges, motivations, and resources associated with this process. To contextualize our Dutch findings, we will subsequently provide an overview of parental sign language courses in the Netherlands. Finally, we will conclude this introductory section by outlining the aims and research questions of this study in more depth.

## Previous work on hearing parents’ experiences with sign language learning

### Family language policy in DHH-hearing families

Following the identification of hearing loss in their child, parents face the complex task of navigating various communication and language choices. This decision-making process can be understood within the framework of Family Language Policy (FLP), defined as “explicit and overt decisions parents make about language use and language learning as well as implicit processes that legitimize certain language and literacy practices over others in the home” (Fogle, 2013, p. 83). In multilingual families, FLP is shaped by various factors, including parents’ skills in each language, family structure, acculturation, and the surrounding language environment (Schwartz, 2010). Parents of DHH children face similar decisions, often at a very early stage in the child’s life (Crowe et al., 2014).

For most hearing parents of DHH children, communication choices include a spoken language, a signed language, or hybrid approaches, such as sign with speech support or speech with sign support. All of these choices require parents to learn new skills and adapt family communication practices to ensure that interactions are fully sensorily accessible to the DHH child (McKee & Smiler, 2016). Research on the linguistic environments of DHH children in Norway shows that most parents adopt a range of communication strategies with their children, adjusting their language use based on the setting, the presence of other adults, and the purpose of the interaction (Arnesen et al., 2008). This reflects broader findings in DHH-hearing families, including families with DHH parent(s) and hearing children, where communication is also typically multimodal and fluid, for instance through sign-speaking and by switching between or blending modalities (De Meulder et al., 2022).

Importantly, FLP is not static. Parents’ decisions often shift over time in response to the DHH child’s linguistic progress and different life stages (Flaherty, 2015) or to parents’ change in views on deafness and disability (Harris et al., 2021). Children also largely influence FLP, for example, by requesting a specific communication mode or by refusing to respond in a certain language or modality (Gafaranga, 2010). When children prefer using the majority language (e.g., a spoken language) over the minority language (e.g., a sign language), this often triggers a shift in FLP over time (Borghetti et al., 2025; Gafaranga, 2010).

### Sociopolitical and ideological influences on Family Language Policy

Parental decisions on FLP are also influenced by broader political and sociocultural pressures, parents’ own perspectives and experiences regarding (sign) languages, as well as additional factors such as a child’s hearing level, cognitive and linguistic abilities, and the use of hearing aids or cochlear implants (Mitchiner & Batamula, 2021). Additionally, parents’ hopes regarding their child’s academic success, social inclusion, and well-being—along with the lived experiences of other parents and DHH adults—play a crucial role in shaping FLP (Harris et al., 2021; McKee & Smiler, 2016).

### The role of professionals in shaping communication choices

Parents frequently experience uncertainty about language choices and often turn to professionals for advice and guidance (Young, 2010). However, communication options are often presented in binary terms—sign language versus spoken language—despite research showing that most parents adopt flexible, combined, or changing strategies over time (Flaherty, 2015; Harris et al., 2021). Moreover, professional advice is often inconsistent. Many professionals prioritize spoken language development and position sign language as secondary or temporary (Chen Pichler, 2022; Mitchiner & Batamula, 2021), and in some cases, discourage its use entirely (Humphries et al., 2012). In New Zealand, for example, studies have documented patterns of professional advice against sign language use following cochlear implantation—even when children had used sign language prior to surgery (McKee & Vale, 2014). Similar findings have been reported in Norway (Kermit, 2010), Australia (Willoughby, 2015), India, Brazil, and the United States (Friedner, 2024; Friedner & Block, 2022).

Humphries et al. (2024) explain that the current narratives among professionals about the role of sign language in a DHH child’s development are so varied that they greatly contribute to parents’ frustration and confusion over communication choices. Interviews conducted by McKee and Smiler (2016) with New Zealand families similarly revealed that parents received conflicting and biased advice, which led to confusion within families, as different family members preferred—or rejected—different communication options. Australian parents interviewed by Harris et al. (2021) also reported receiving partial or biased information and noted that conflicting views from different service providers about what was “best” for the DHH child made the decision-making process highly complex, uncertain, and confusing.

### Parents’ motivations for learning sign language

Research on hearing parents who choose to learn sign language highlights a variety of motivations for doing so. For instance, Lieberman et al. (2024) surveyed 100 hearing parents of DHH children aged 3 to 21 about their motivations for learning ASL. The most commonly cited reason was “to have a relationship with their child by communicating effectively with them” (p. 17), followed by “to support the belief that ASL is the most natural and accessible language for their deaf child” (p. 17). Parents thus saw learning ASL as an opportunity to provide their child with full access to a natural language. Other important motivations included supporting their child’s development and identity, as well as being part of the DHH community.

### Parents’ challenges in accessing and learning sign language

Despite strong motivations, parents face numerous challenges in accessing appropriate sign language instruction. One major issue reported in studies on ASL and NGT is the limited availability of courses—particularly at advanced levels or tailored to the needs of parents with older children (Lieberman et al., 2024; Oyserman & de Geus, 2021). Institutional support is often lacking. Research on New Zealand Sign Language, for instance, highlights that parents often consider guidance, resources, and learning opportunities weak or even actively discouraging (McKee & Smiler, 2016).

Another common challenge is that most sign language courses are designed for adults in general rather than specifically for parents. As a result, parents often learn how to communicate with other adults rather than young children (Napier et al., 2007; Oyserman & de Geus, 2021; Pontecorvo et al., 2024). Many parents also feel there are insufficient self-study materials available for hearing families (Lieberman et al., 2024). Furthermore, most courses rely heavily on the written form of the majority language, which creates barriers for parents with a different language background (Willoughby, 2009, 2015).

Linguistic challenges also present a barrier for hearing parents learning sign language. Many parents cite the general difficulty of learning a new language as an adult (Dutra, 2020; Lieberman et al., 2024; Napier et al., 2007). In the context of learning a sign language, parents often report struggling with mastering sign language grammar and forming sentences (Chen Pichler, 2021; Decker & Vallotton, 2016). While many rely on sign language dictionaries for learning (Lieberman et al., 2024), dictionaries primarily aid in expanding vocabulary but provide little support for learning to form sentences effectively. Zarchy and Geer (2023) found that although families often learn a lot of ASL vocabulary on their own, these are predominately nouns and pre-academic words (e.g., colors, animals, and clothing) rather than function words essential for sentence construction. Even in formal ASL courses, parents frequently express frustration over the lack of instruction on grammar and language structure (Chen Pichler, 2021; Decker & Vallotton, 2016). Similarly, Oyserman and de Geus (2021) reported that while Dutch parents had learned a substantial amount of vocabulary during their NGT courses, they barely learned the syntactical, semantic, and morphological features of NGT.

In addition to linguistic challenges, external factors such as limited time and financial resources often make it difficult for parents to attend sign language courses (Dutra, 2020; Lieberman et al., 2024). For families in rural areas, long travel distances can pose an additional barrier (Lieberman et al., 2024). In some cases, families even choose to relocate in order to access resources such as DHH schools or larger DHH communities, or quit their jobs to create time for sign language learning—something that is not financially feasible for most parents (Chen Pichler, 2021). A lack of opportunities to interact with DHH individuals further restricts immersion in the language and culture (Lieberman et al., 2024; Napier et al., 2007). Supportive policies, however, can help address these barriers. In Norway, for example, parents are entitled to ~1,600 hr of sign language instruction spread over the child’s first 16 years. Parents also receive paid leave from employment, and room, board, and transportation are provided (Arnesen et al., 2008; Haualand & Holmström, 2019). These measures reduce financial and time constraints and allow parents to continue learning as their child grows older.

Another external obstacle, as mentioned earlier, is societal bias and prejudice against sign language. Many parents encounter and are influenced by misinformation from medical professionals, who often falsely claim that sign language will hinder their child’s spoken language development (Dutra, 2020; Humphries et al., 2024; Lieberman et al., 2024; Mitchiner & Batamula, 2021; Willoughby, 2012). Acceptance of their child’s hearing status can also play a crucial role in shaping parents’ learning experiences (Dutra, 2020; Willoughby, 2009, 2012). Dutra (2020) found that all 10 parents interviewed described this acceptance as a key part of their ASL learning journey. These findings illustrate that parents’ experiences with sign language learning are shaped by a complex interplay of personal perspectives, societal ideologies, linguistic barriers, and broader structural conditions.

## Parental sign language courses in the Netherlands

This section explores parental sign language courses in the Netherlands. It begins by outlining the general process for hearing families to access these courses and proceeds to describe their general organization.

### Access to courses

In the Netherlands, hearing loss in children is typically identified shortly after birth through newborn hearing screening. If a child’s screening results indicate potential hearing loss, the family is referred to a speech and hearing center for audiological assessment (Van der Zee et al., 2022). Based on this assessment, the center then connects parents with specialized organizations^1^ that provide family-centered early intervention (FCEI) for DHH children. FCEI supports parents in fostering their DHH children’s language development through care and (in-home) guidance, which potentially includes parental sign language courses.

The type and number of sign language courses offered as part of FCEI depend on the specialized organization assigned to the family, which is based on the family’s geographic region (Van der Zee & Dirks, 2022). Parents typically cannot switch organizations, nor can they access courses offered by other organizations. As a result, the availability of sign language courses is ultimately determined by the family’s location—resulting in a postcode lottery of support. For families in rural areas, this often means fewer available courses—if any—and longer travel distances to attend them.

Eligibility for FCEI, including parental sign language courses, is limited to children identified with permanent bilateral hearing loss of ≥40 dB (Van der Zee & Dirks, 2022). To access the courses, parents must have a referral from a doctor or audiologist. While health insurance theoretically covers the costs^2^—reducing the financial burden on parents—the funds are allocated directly to the specialized organizations. As a result, parents are dependent on the organization’s course offerings and communication. Additionally, health insurance providers may limit the number of courses, often covering only basic courses or requiring an out-of-pocket contribution from parents. Although NGT resources offered outside specialized organizations are available, such as online courses, learning platforms, and apps, these are generally not eligible for insurance reimbursement. This is because sign language instruction for parents is classified as part of the healthcare system. Only providers with official healthcare provider status—the specialized organizations mentioned above—can receive insurance funding. Independent teachers or institutions without this status cannot have their services reimbursed, even if parents prefer them. As a result, this healthcare structure limits parental choice and flexibility in accessing sign language instruction.

Parental sign language courses typically end when the child reaches the age of 4. This age limit is not based on language development needs, but on the structure of the healthcare system: sign language instruction for parents is part of the temporary FCEI care trajectory, which often ends when children begin formal schooling. As a result, there are few options for parents with older children—regardless of whether families started with sign language early or only decided to pursue it later in the child’s life. Studies on NGT also emphasize the limited availability of advanced sign language courses for parents with older children (Oyserman & de Geus, 2021). After the FCEI period, families must request a specialized care trajectory to continue accessing insurance-covered sign language instruction.

### Course structure

While the course structure varies between specialized organizations, initial courses are often conducted in the family home, allowing other family members or caregivers to participate. Later courses are typically held in group settings with other parents of DHH children at the organization’s premises. The type of sign language instruction provided also differs between organizations. Some offer courses in NGT, while others focus on Sign-supported Dutch (*Nederlands met Gebaren*, NmG). NmG combines spoken Dutch with keyword signing, aiming to make spoken language more visually accessible. However, in practice, NmG often results in a poor representation of both Dutch and NGT, making it difficult to understand for those with limited or no sensory access to spoken language (De Meulder et al., 2019; Terpstra & Schermer, 2006).

Since parents are assigned to an organization based on their geographic region, they have little choice in the type of instruction they receive. This regional assignment largely determines whether families learn NGT or NmG, as organizations differ in their language ideologies and teaching approaches.^3^ Additionally, some organizations offer courses that blend NmG with elements of NGT grammar, creating a continuum between Dutch and NGT, with NmG positioned at varying points along this spectrum. In some cases, instructors even adapt NmG courses to include more NGT upon parental request, although they may still rely on official NmG materials provided by the organization. Consequently, geographic location significantly shapes families’ experiences and opportunities for learning.

Regardless of whether the course focuses on NmG or NGT, instructors include both DHH and hearing professionals. This is because the Netherlands has one official teacher training program for NGT, open to both hearing and DHH students, with hearing students making up the majority of graduates. Additionally, both NmG and NGT courses rely heavily on (written) Dutch in their teaching materials, which can create barriers for parents with a different language background.

Finally, organizations do not always develop their own sign language materials and often rely on course content managed by external entities. Because these materials are owned by external entities, access can be restricted, making specialized organizations dependent on whether and how these resources are shared. As a result, some organizations may end up using outdated materials due to limited access to updated versions.

Overall, the organization of parental sign language courses in the Netherlands reflects the interplay between geographic location, the access policies set by healthcare providers and specialized organizations, and the language ideologies of these organizations regarding NGT and NmG instruction.

## Current work

Several studies have examined the sign language learning experiences of hearing parents, as outlined in the introduction. These studies highlight the limited availability of sign language courses and resources, which tend to focus primarily on vocabulary rather than sentence construction. Additionally, external challenges such as limited time and money for courses, as well as negative attitudes toward sign language, have been identified. However, much of the existing research is centered around hearing parents learning ASL in the United States.^4^ To gain a broader understanding of these experiences, it is important to consider a wider range of sign languages and cultural and sociopolitical contexts. Given that the availability and quality of sign language courses and resources vary across countries and depend on national parental support systems, we expect to find country-specific differences in parents’ experiences. This research aims to deepen our understanding of hearing parents’ sign language learning experiences by including qualitative data from Dutch parents and NGT teachers who teach parental courses.

To explore these experiences, we conducted semi-structured interviews to address the following research questions:

1. How do parents and teachers evaluate sign language courses?
2. What are parents’ and teachers’ views on supplementary sign language materials?
3. What general challenges do parents face while learning sign language?

Through these questions, we intend to (1) examine parents’ experiences with the sign language courses they attended and teachers’ perspectives on the parental courses they delivered, (2) gather insights on supplementary resources—such as sign language apps, online platforms, and dictionaries—used in addition to the course materials, and (3) explore the specific challenges Dutch parents face in sign language learning.

## Method

This research study was approved by the Ethics Committee of the Faculty of Humanities of the University of Amsterdam. The study consisted of semi-structured interviews with two participant groups: (1) hearing parents of DHH children and (2) Dutch Sign Language (NGT) teachers who teach hearing parents of DHH children. While this study’s aim was twofold, namely, to understand parents’ sign language learning experiences in the Netherlands and to gather feedback on an NGT learning app we are developing, this paper focuses only on exploring parents’ learning experiences. This study draws on data from both parents and NGT teachers to explore parental learning experiences from multiple perspectives.

### Participants

#### Eligibility and recruitment

Hearing parents of DHH children were eligible to participate in this study if they were currently enrolled in or had previously completed a NGT or Sign-supported Dutch (NmG) course. All but two parents in our sample attended parental sign language courses offered by specialized organizations, as described in the introduction, with some also pursuing additional courses outside these organizations, such as university classes or interpreter training programs. The remaining two parents participated in courses provided outside early-intervention programs. NGT teachers were eligible to participate if they had experience teaching NGT to hearing parents of DHH children.

There were no age restrictions for the DHH children of the parent participants, which helped increase the number of participants and allowed for a wider range of experiences from parents with children of varying ages. This approach aimed to capture both the current experiences of parents with young children who still followed parental sign language courses as well as the reflections of parents with older children on their sign language learning journey. Including both NGT and NmG learners was intended to attract a larger and geographically diverse participant pool, as NmG courses are standardly offered in some regions of the Netherlands.

Participants were recruited through social media sites directed at parents of DHH children, organizations serving parents of DHH children—including the Royal Dutch Auris Group, the Dutch Federation of Parents of Deaf Children (FODOK), the mental health and social services for the DHH (GGMD), and Royal Dutch Kentalis—and personal contacts. Participants received a 20-euro gift card upon participation.^5^

#### Parent demographics

Twenty-two parents participated in this study. However, data of one parent were excluded from the results due to their child’s age differing substantially from that of the other participants’ children—by 12 years above the sample mean—which meant the parent had taken sign language courses over a decade ago and therefore could no longer fully recall their learning experiences. In the end, 21 parents (17 female, 4 male) comprising 20 different families were included. The mother and father from the same family were interviewed together, while all other participants were interviewed individually. The gender imbalance in our sample may reflect a broader trend observed during in-home early-intervention sessions, where mothers tend to participate more frequently than fathers (Dirks & Szarkowski, 2022).

Participants were geographically diverse, residing in seven different provinces across the Netherlands, representing the southern, central, and northwestern regions. Most parents had one DHH child, though one parent had two DHH children. The ages of the children ranged from 2 to 9 years (mean = 6 years). All parents were hearing, except for one parent who was hard of hearing. While the participant call targeted hearing parents, this hard-of-hearing parent was included due to their lack of prior experience with sign language before their child’s hearing loss was identified.

When asked about the type of courses they had taken, nine parents reported attending NGT courses, seven reported NmG courses, and five had attended both NGT and NmG courses. This information was gathered to explore whether the type of course influenced parents’ learning experiences, particularly regarding challenges with grammar and sentence structure. However, as discussed in the section on course structure, the distinction between NmG and NGT courses is not always clear-cut, as NmG can be seen as existing on a continuum between Dutch and NGT. Therefore, this distinction should be interpreted with caution. Table 1 below summarizes parent demographics.

**Table 1:** Summary of parent demographics

	Overall (*N* = 21)
*Parent*	
Mother	17
Father	4
*Age of DHH children (years)*	
Mean (*SD*)	5.57 (2.31)
Median [min, max]	6.00 [2.00–9.00]
*Hearing status*	
Hearing	20
Hard-of-hearing	1
*Type of course*	
NGT	9
NmG	7
NGT and NmG	5

*Note*. DHH = deaf and hard-of-hearing; NGT = *Nederlandse Gebarentaal*; NmG = *Nederlands met Gebaren*.

This study did not collect information about parents’ educational background, socioeconomic status, or ethnicity.

#### Teacher demographics

Six NGT teachers participated in this study, each of whom was interviewed individually. Of the six teachers (four female, two male), three were hearing and three were DHH. This balance in participants’ hearing status was coincidental rather than planned. Two of the teachers—both DHH—considered NGT as their mother tongue. All teachers were rather experienced, with a mean of ~15 years of NGT teaching experience (range: 3–27 years), including teaching to groups other than parents. Table 2 summarizes teacher demographics.

**Table 2:** Summary of teacher demographics

	Overall (*N* = 6)
*Gender*	
Female	4
Male	2
*Hearing status*	
DHH	3
Hearing	3
*First language(s)*	
Dutch	3
Dutch and Frisian	1
Dutch and NGT	1
NGT	1
*Teaching experience (years)*	
Mean (*SD*)	14.83 (8.23)
Median [min, max]	14.50 [3.00–27.00]

*Note*. DHH = deaf and hard-of-hearing; NGT = *Nederlandse Gebarentaal.*

### Interview structure

Interviews were conducted by the first author between February and May 2024 at a location of the participant’s choice, typically involving home visits. The first author is a hearing, native Dutch speaker who learned NGT as an adult, starting in 2020, and has used NGT on a daily basis in interactions and work meetings with DHH colleagues since 2023. Interviews lasted 1 hr and 15 min on average (range: 34 min to 2 hr and 15 min) and were conducted in Dutch, except for two interviews held in NGT. All interviews were audio recorded, with the NGT interviews additionally being video recorded. In most cases, only the researcher and participant(s) were present, though occasionally children or non-participating partners were also present in the background.

For the NGT interviews, a sign language interpreter was present, selected based on the participant’s preference. The researcher and participant communicated directly in NGT, with the interpreter simultaneously translating all interactions from NGT into Dutch throughout the interview. This ensured that the researcher could cross-check her understanding while maintaining direct communication in NGT. While the presence of an interpreter influences the interaction,^6^ the decision was made to have an interpreter present to reduce the risk of misunderstandings or missing details in the participant’s responses.

Informed consent was obtained from all participants before the start of the interviews. The interview started with demographic questions, after which the rest of the interview was divided into two parts. In the first part, participants were asked about their experiences with NGT/NmG courses, including descriptions of the courses they attended or taught, positive aspects, and areas for improvement. They were also asked for ideas on how to complement these courses. Parents were then asked about supplementary materials used for self-study, while teachers were asked about additional materials used during their classes alongside the standard course materials. Finally, participants discussed general challenges in learning NGT/NmG. Appendix 1 provides a complete list of the prepared interview questions.

The second part of the interview focused on gathering feedback on a prototype of our NGT app. As this is not the focus of the current article, this part of the interview will not be further described in the current paper.

### Coding and analysis

All interviews were transcribed automatically using Whisper AI software. Transcripts of the Dutch interviews were manually checked against the original audio recordings, while transcripts of the interviews conducted in NGT were verified against the original video recordings. The verified transcripts were then coded in ATLAS.ti, with the initial coding aimed at identifying information relevant to our research questions (see section *Current Work* on pages 10–11). During this stage, the following codes were used: *GeneralChallenge*, *Course_positive*, *Course_negative*, C*ourse_HowToComplement*, *AdditionalMaterial_which*, *AdditionalMaterial_positive*, and *AdditionalMaterial_negative*. Additionally, the code *LanguageOptions* was added as discussions about language and communication choices—whether to use NGT, NmG, or spoken Dutch at home—emerged prominently, even though this was not an initial research focus.

After initial coding, all coded segments were further manually coded to account for the different challenges, positives and negatives. For instance, segments coded as *GeneralChallenge* were again coded with focused codes, such as *lack of time*, *perceived difficulty with the language*, and *general difficulty in learning a new language*, to account for the specific challenges parents experienced*.* After all coding was complete, the focused codes were counted to determine in how many interviews each code appeared.

## Results

This section presents the findings from interviews with parents learning Dutch Sign Language (NGT) or Sign-supported Dutch (NmG) and NGT teachers involved in parental courses. It examines the strengths and areas for improvement in these courses, the use of supplementary materials, and the general challenges parents face when learning sign language. Additionally, the results highlight the perspectives of both parents and NGT teachers on choosing or balancing spoken Dutch, NmG, and NGT in communicating with their DHH children.

Parents learning NGT and NmG are presented as a single group in the analysis. This decision was based on the difficulty of reliably distinguishing between these two groups. Many parents in the sample participated in courses described by their respective organizations as NmG with elements of NGT. Furthermore, during the interviews, it became evident that parents were not always aware of the differences between NmG and NGT. In many cases, parents learning NmG demonstrated a substantial understanding of NGT grammar. Although a distinction between NmG and NGT could potentially reveal interesting differences, it was not feasible to create such a distinction in a meaningful way because of the difficulty of analytically separating the two. While there is a clear experiential and cognitive impact of NGT versus NmG on DHH children who are perceiving it, NmG, as used in educational and parental contexts, should be understood as a socially constructed category shaped by specific linguistic and cultural ideologies (De Meulder et al., 2019) rather than as an objective division.

### Course experiences

Parents reported numerous positive aspects of the NGT or NmG courses they had taken or were still enrolled in. The most frequently mentioned benefits included having a DHH teacher—providing both immersion into the language and a valuable role model—achieving good course results and having initial courses taught at home. Having lessons at home was not only convenient since it reduced travel time and the need for babysitters, but it was also seen as a very comfortable way to start learning the language. Several parents described signing as a hurdle at first, something they had to overcome.^7^ They felt self-conscious when beginning to sign, and appreciated the privacy and familiarity of their home setting during this initial phase. Besides, it made it easier for other family members to join classes, which was seen as a large positive.

One parent reflected on their experience with DHH teachers:

Well, yes, I have to think a lot about both ^*^names of two teachers^*^, they were our first trainers or teachers. They are both hard of hearing from birth and both diagnosed late. And I know that really reassured me a lot. They are both nice people, so that… You quickly get the idea, you know, the child is disabled, or there’s something. And it was partly because of them that I actually thought, well, there's nothing wrong at all. We can't make any mistakes, because it will be okay. (Author’s translation)

This parent’s experience aligns closely with findings from a New Zealand study by McKee and Smiler (2016), who conducted interviews with hearing parents of DHH children and found that encounters with DHH individuals helped reduce fears about their child’s future. They note that when a DHH interlocutor’s characteristics align with the family’s own identity and aspirations—for example, in ethnicity or perceived competence (e.g., “married,” “been to university”)—that individual can become a meaningful role model for the family, helping them interpret information and envision possible futures for their child.

Parents also appreciated the online learning platforms provided as part of some courses. They noted that these platforms featured high-quality videos, were well structured, and provided the opportunity to repeat learning material at their own pace and time. Other positive aspects of the courses included good teachers, whom parents often described as pleasant, highly experienced, or knowledgeable. Additionally, parents highly valued the alignment of course content with their child’s life stage—making it directly applicable to daily life—and the opportunity to connect with other hearing parents of DHH children during group lessons. Table 3 provides a complete overview of the strengths reported in at least a quarter of the interviews.

**Table 3:** Most commonly reported strengths of NGT/NmG courses by parents (*N* = 20)^14^

Code	Number of interviews mentioning code
Deaf or hard-of-hearing instructor^15^	8
Course taught at home	8
Positive learning outcomes	8
Inclusion of an online learning platform	7
Pleasant or skilled instructor	7
Content aligned with child’s age or developmental stage	6
Contact with other hearing parents of DHH children	6
Opportunity for family involvement	6
Tailored to individual needs	5

*Note*. DHH = deaf and hard-of-hearing; NGT = *Nederlandse Gebarentaal*; NmG = *Nederlands met Gebaren*.

^14^Throughout this paper, *N* refers to the number of interviews.

^15^In two interviews, having a DHH teacher was mentioned as a positive as well as a downside since these parents felt it made asking questions more difficult.

Several points for improvement were also identified. Parents often noted that the course content was aimed at children of a different age, making it difficult to apply the material at home and leading to forgetting the signs they had learned. This misalignment often occurred when parents began courses later in their child’s development or simply enrolled as soon as courses became available, fearing they might otherwise miss critical opportunities.

Another frequently mentioned downside was that the course level was too basic, with parents perceiving the courses as lacking challenge or depth and progressing too slowly. Although previous literature shows that hearing parents often perceive sign language as difficult to learn (Chen Pichler, 2021; Decker & Vallotton, 2016), parents explained that this issue often stemmed from mixed language levels among participants, with some progressing more slowly and teachers adjusting the pace to accommodate them.

Parents further highlighted the absence of explicit grammar instruction, outdated materials, and an insufficient number of lessons. Many felt that the lessons were too infrequent or the courses too short, leaving them feeling not fully self-reliant after the course ended. These issues were mentioned in at least a quarter of the interviews, as shown in Table 4.

**Table 4:** Most commonly reported points for improvement of NGT/NmG courses by parents (*N* = 20)

Code	Number of interviews mentioning code
Content misaligned with the child’s age	7
Course progresses slowly or includes excessive repetition	7
Differences in skill levels among participants	7
Outdated materials	6
Course lacks challenge or depth	5
Need for more or earlier instruction on grammar	5
Insufficient number of lessons	5

*Note*. NGT = *Nederlandse Gebarentaal*; NmG = *Nederlands met Gebaren*

When asked how courses could be complemented to address these gaps, ideas were rather diverse. In five interviews, parents mentioned a sign language learning app as a way to complement courses. However, participants were aware of the NGT app we are developing and knew they were about to try out our prototype version, which may have influenced their responses. They viewed an app as an easy way to look up signs without needing to log in or require a computer and imagined an app as an interactive way to practice sign language at their own pace.

Other suggestions included courses and materials targeted at parents of older children or more advanced learners (noted in four interviews). Additionally, some parents desired more accessible materials without lengthy log-in processes (*N* = 2) and more guidance in connecting with other sign language learners or users (*N* = 2).

Teachers were also asked about the positives and areas for improvement in the sign language courses they taught to parents. Since the teachers were involved in multiple courses, they often identified certain aspects as strengths in one course that they perceived as lacking in another. It is further important to note that, given the small number of teacher participants (*N* = 6) and the use of open-ended questions to inform what they perceived as strengths and areas for improvement, no firm conclusions should be drawn from what was *not* mentioned. A lack of mention does not necessarily indicate a lack of importance; teachers may have shared similar views but simply did not articulate them during the interview. Nevertheless, recurring themes did emerge across the responses.

Half of the teachers valued that some courses extended beyond vocabulary level, included an online learning platform, featured videos of parent–child signing (with both DHH and hearing parents), and used visual rather than text-based materials. The online learning platform was particularly appreciated for offering a way to practice and repeat material outside of class, and teachers found it to be modern, well structured, and interactive. Teachers appreciated the videos of parent–child signing featuring DHH parents, as these provided strong examples of L1 child-directed signing. They also valued the videos of hearing parents, noting that these offered relatable role models and demonstrated that signing does not have to be perfect—as long as parents keep signing.

Other positive aspects mentioned by teachers included content being tailored specifically to parents and the use of game-based exercises to allow for much repetition in a fun, engaging way. Table 5 below provides a complete overview of the strengths reported by at least one-third of the teachers.

**Table 5:** Most commonly reported strengths of NGT courses by teachers (*N* = 6)

Code	Number of interviews mentioning code
Content extending beyond vocabulary level	3
Inclusion of an online learning platform	3
Use of videos featuring parent–child signing	3
Visual materials instead of text-based	3
Content tailored to parents	2
Game-based exercises	2
Order of instruction: in-class instruction first, followed by self-study (homework)	2
Positive learning outcomes	2
Use of modern materials	2

*Note*. NGT = *Nederlandse Gebarentaal*.

Teachers also pointed out several areas for improvement. The most common criticisms included too much focus on vocabulary rather than full sentences and the course containing outdated materials. Additionally, some teachers noted the content not being tailored to parents, a lack of explicit grammar instruction, and an overemphasis on written Dutch or translation. One teacher explains:

*Previously, courses contained more text. I felt like how can a sign language course be in written Dutch? As a result, it became incredibly literal. People started signing very literally. And we really wanted to shift that to using visual images instead.* (NGT interpreter’s translation, validated by participant)

This teacher emphasized that, as visual languages, sign languages require teaching materials that align with their visual nature. Another teacher commented on the focus on vocabulary:

*It’s all very focused on the word level. So that doesn’t make me very happy. Because, I mean, you don’t communicate with your child in words. These will have to become full sentences.* (Author’s translation)

The order of in-class instruction and self-study was also criticized by two teachers as during some courses parents were required to do their homework before rather than after class.^8^ They noted that parents often failed to complete their homework due to lack of time or motivation (as will be discussed in the section *General Challenges* on page 23), leading to difficulty in following the scheduled lesson plan containing further in-class practice with the homework. Moreover, they pointed out that learning vocabulary at home from videos is not ideal for a visual language. They mentioned that parents find it difficult to learn new signs without seeing them produced in person first where the teacher can choose the best angle to show the sign and correct parents if needed. As a result, parents frequently arrived at class having learned signs incorrectly, requiring teachers to spend class time unlearning errors before moving forward. Table 6 below provides a complete overview of the most commonly mentioned points of improvement by teachers.

**Table 6:** Most commonly reported points for improvement of NGT courses by teachers (*N* = 6)

Code	Number of interviews mentioning code
Lack of sentence practice/Too much focus on vocabulary	4
Outdated materials	4
Content not tailored to parents	2
Materials being no longer accessible	2
Need for more or earlier instruction on grammar	2
Order of instruction: self-study (homework) first, followed by in-class instruction	2
Text-based materials instead of visual	2
Overemphasis on Dutch or translation	2

*Note*. NGT = *Nederlandse Gebarentaal*.

Ideas for complementing existing courses varied, with half of the teachers wishing for more or richer NGT material at different proficiency levels. This would provide the teachers with more practice material to choose from. Two teachers also expressed a desire for more NGT-based games.

A comparison between the perspectives of parents (Tables 3 and 4) and teachers (Tables 5 and 6) reveals a notable difference in focus. Parents primarily named external or contextual factors as strengths, such as having a DHH teacher, receiving instruction at home, and having opportunities to connect with other parents of DHH children. These responses suggest that parents place a high value on the broader learning environment and social–emotional support.

In contrast, when identifying points for improvement, parents shifted their focus to the content and delivery of the course itself, mentioning issues such as misalignment with their child’s developmental stage, slow progression, outdated materials, and insufficient grammar instruction. This indicates that parents mostly valued the external and contextual aspects of their learning experiences, while most of their points for improvement centered around the course itself.

Teachers, on the other hand, consistently focused on course content and structure in both their positive and critical responses, such as the inclusion of videos, the use of visual rather than text-based materials, and the ordering of instruction.

This divergence likely reflects the different roles and priorities of each group: while parents are mainly focused on course accessibility and emotional support, teachers focus more on effective teaching and resource quality. Notably, both groups mentioned positive learning outcomes and the inclusion of an online learning platform as strengths, and both identified outdated materials and the need for more or earlier instruction on grammar as points for improvement.

### Use of supplementary material

Parents used a variety of resources for self-study, with the online NGT dictionary^9^ being the most frequently mentioned (*N* = 16). Many parents received a 1-year complimentary subscription to the dictionary during their courses, which they also used for self-study alongside or after completing their course. Additionally, parents used various apps and online platforms.^10^ Interestingly, in three-quarters of the interviews, parents named online materials designed for children as learning resources. YouTube also appeared to be a popular tool for learning and retrieving signs (*N* = 8).

When asked about the benefits of these supplementary materials, parents highlighted several positive aspects. Many appreciated that these resources were enjoyable for their child and could be used together as a shared activity. Several parents were amazed to see their child engaging with and enjoying sign language, as watching these materials often formed the child’s first exposure to sign language. Half of the parents mentioned that the additional materials were fun or engaging, serving as a motivating way to learn.

Other strengths included positive learning outcomes, easy sign retrieval, and content aligned with their child’s life stage. In six interviews, parents reported using additional materials, such as social media platforms or television shows with interpreters, to keep sign language active in their daily lives. Since parents lacked regular exposure to sign language, they actively sought ways to surround themselves with the language through these resources.

Finally, parents valued the low-pressure nature of these materials, describing them as an informal, accessible way to practice sign language at their own pace and time. Table 7 summarizes the most commonly reported strengths of self-study materials.

**Table 7:** Most commonly reported strengths of self-study materials by parents (*N* = 20)

Code	Number of interviews mentioning code
Child enjoys it/Can be used together with child	12
Fun	10
Positive learning outcomes	9
Provides sign language exposure/Way to keep sign language active	6
Easy sign retrieval	6
Content aligned with child’s age	5
Low-pressure learning setting	5

Parents identified several areas for improvement in the self-study materials they used. The most commonly reported issue was limited content or a desire for more elaborate material (*N* = 14). In the case of online learning platforms and apps, parents often wished to continue learning for a longer duration. For the online dictionary and vocabulary apps, many parents reported not being able to find the signs they needed. Other challenges included materials not being easily accessible, either because of non–user-friendly interfaces (mainly related to lengthy log-in processes) or because the materials were only accessible via computer and not also on a phone. Associated costs and the materials being too advanced in terms of sign language level were also seen as disadvantages, see Table 8.

**Table 8:** Most commonly reported points for improvement of self-study materials by parents (*N* = 20)

Code	Number of interviews mentioning code
Limited content/Desire for more elaborate material	14
Only available on computer/No app available	8
Not user-friendly	7
Associated costs	6
Signing produced too quickly/Material is too advanced	6

Teachers were asked which additional materials they used during their lessons alongside the standard course material. These materials thus generally differed from the supplementary materials mentioned by parents, as these materials were used by teachers for providing extra practice or examples during class rather than being aimed at self-study. The most used resources included the online NGT dictionary, self-made materials, and materials from other courses (all noted in four interviews). Self-made materials included a large variety of things, such as exercises based on collected photos or images, self-composed vocabulary lists, and self-fabricated flashcards, games, and puzzles used for NGT learning. Other supplementary resources included videos from the NGT corpus^11^ (*N* = 3), NGT-based games (*N* = 3) such as memory matching games and quartets, and YouTube videos (*N* = 2).

Strengths of these materials included them being enjoyable for children, which facilitated parent and child learning together. Additionally, some materials provided handshape information by depicting a handshape icon next to the sign’s video or offered exposure to different NGT signers and input.

Disadvantages included the materials being too advanced for the course level, issues with user-friendliness due to confusing or lengthy log-in processes, and the self-made materials being very time consuming to create.

When comparing parents’ and teachers’ perspectives on additional learning materials, several similarities and differences emerge. Parents and teachers both identified the online NGT dictionary as the most commonly used additional resource for learning. Beyond this shared tool, the additional materials mentioned largely differed, reflecting their use in different contexts—self-study by parents versus use during classroom instruction by teachers. Interestingly, both groups viewed materials that are enjoyable for children as a major strength (see Tables 7 and 9). Parents often expressed excitement at seeing their children engage with sign language, while teachers viewed this as a way to encourage joint learning between parent and child.

**Table 9:** Most commonly reported strengths of additional materials by teachers (*N* = 6)

Code	Number of interviews mentioning code
Enjoyable for children/Can be used together with child	2
Provides information on signs’ handshapes	2
Exposure to different NGT signers	2

*Note*. NGT = *Nederlandse Gebarentaal*.

**Table 10:** Most commonly reported points for improvement of additional materials by teachers (*N* = 6)

Code	Number of interviews mentioning code
Material is too advanced	2
Not user-friendly	2
Time-consuming to create own materials	2

As for points for improvement, both parents and teachers noted that many materials are too advanced for parents’ current learning levels. Additionally, both groups mentioned a lack of user-friendliness, mostly referring to the online NGT dictionary.

### General challenges

Parents described several challenges in learning NGT/NmG. Most struggled with specific components of the language, such as NGT grammar and sign order, accurately producing all sign parameters (handshape, location, orientation, movement, and non-manual features), and adjusting to a visual language. In addition to these language-specific difficulties, several external challenges were also mentioned. In nearly half of the interviews, limited availability of suitable courses as well as difficulty in gaining access to these courses were mentioned as a struggle. Several parents mentioned that they were very motivated to start learning but they felt thwarted by their assigned specialized organization or healthcare provider that did not provide sufficient or correct information, or that did not see the need for them to learn sign language. Parents mentioned a lot of insurance paperwork and money issues when it came to accessing courses. One parent reflected:

*Well, getting access to the courses was really intense. I think that, in those first years, we spent more time in meetings and asking and fighting for sign language courses. All those emails, all those contacts, switching to another healthcare provider who in turn worked with subcontractors. And well, figuring all of that out, talking to people on the phone who had no idea what the difference is between NmG and NGT. […] Oh, it’s such a struggle. And yes, once you finally understand it all, everything seems easier, but it takes you two or three years or so, with countless conversations and half-information or wrong information, just to be able to take a sign language course. It’s absurd. It takes an awful lot of time, and if you could just put all that time into learning to sign, you’d already be so much further along.* (Author’s translation)

Several parents also missed guidance in the process and mentioned not knowing how to start learning sign language. One parent explained:

*And I remember that we really pushed for it back then. We had no idea how to start. I mean, what did we know, right, learning a new language—no idea how to go about that. We really pushed for it, like ‘We would like to START, START, START with… with Kijk ik Gebaar* [name of NGT course]’*. But it didn’t really get off the ground. They also told us at first, you know, ‘It isn’t very useful yet, just start with a few signs’. And eventually, in August 2022, when he was, what, about 7 months old, we started with Kijk ik Gebaar 1* [name of NGT course]*. But for us, it actually felt… way too late.* (Author’s translation)

Others described feeling that their urgency to learn sign language was not acknowledged by professionals. As one parent expressed:

It’s actually very bad because as parents, you almost have to beg to be able to learn sign language. […] Well, we were fully committed, because we could see… Right away you could see them [people at daughter’s early intervention group] using sign language and how beneficial it was for ^*^child’s name^*^. But as parents, you really feel the lack of them understanding that it’s also very important for us to learn so that we can use it at home. Actually, we really felt like it should just happen as fast as possible, like getting lessons every day. But, well, that’s not how they see it. (Author’s translation)

Many parents reported that their journey with NGT/NmG began during an especially challenging period of life. At this time, they were often managing the demands of caring for a young child, processing the news of their child’s hearing loss, navigating intense cochlear implant procedures, or feeling isolated due to not knowing other parents of DHH children. Additionally, parents expressed frustration about the lack of advanced courses once their children grew older. They described wanting to learn more NGT/NmG but that courses were only offered at a basic level and did not continue after their child’s age of 4 or 5. Parents felt left in the dark once they completed these courses as they did not have the signing skills to communicate effectively as their child’s language and cognitive abilities developed. One parent with a young hearing child and a DHH older child explained:

*It’s just so frustrating that you just… don’t speak your child’s mother tongue. And that goes completely against your own intuition. […] I can now really see the difference between the oldest and the youngest. With the youngest, it’s so easy. I can explain anything I want to her. I can tell her anything. I can easily express my anger towards her. I can easily show my happiness. I can express all my emotions to her very clearly, without having to make any effort. And with my oldest child… It’s already very difficult, for example, to get angry. And if you want to get angry, it’s hard to make clear why you’re angry. It’s really very difficult. I’ve really struggled with that. And it’s not that I want to be angry with her all the time. But you also want to set certain boundaries for her. Or certain things… For example, when you’re really happy about something or very proud of something she does, you also want to be able to express that. And that just isn’t fully possible yet. […] And very often, it breaks your heart, actually. Because you just… yeah.. want to fully provide her the language that you master well. But yes, it just doesn’t work.* (Author’s translation)

In seven interviews, parents named using sign language at home or in daily life as a challenge. They mentioned falling back on spoken language because it was easier in the moment, because of difficulty balancing between spoken and sign language within their hearing family, or because their DHH child did not want them to sign. One parent described how their child initially resisted sign language but later became open to it:

*What I also noticed was… My son had a strong aversion to sign language in the beginning. So that was quite a challenge. Because I was very motivated. I also thought it would be really fun to learn. But he had a kind of aversion to it. So he was like ‘You should just talk to me’. And yeah… try signing, then. Because I was definitely very driven in the beginning. Like, well I’m going to use sign language. But he was like… ‘Go away, I don't want any of this’. I think he also doesn’t really want to be deaf—who does? But well, yeah… I think he went through a sort of internal battle with it. And now he's at a special school for deaf and hard-of-hearing children. And there, they do nothing else. And now he can actually sign quite well. […] Now he thinks, okay, I can do this. I feel good about this.* (Author’s translation)

This parent noted that their child’s attitude toward signing shifted after enrolling in a DHH school where signing is the norm. The child’s willingness to sign appears to be shaped by their environment and by perceptions of what is considered normal.

In a quarter of the interviews, negative attitudes or opinions by others on sign language use were mentioned. Parents expressed being stared at on the street while signing, as well as professionals, such as speech therapists, medical doctors, and itinerant practitioners,^12^ giving unasked-for, incorrect, or conflicting advice regarding the use of sign language. One parent shared:

*Because he was only deaf in one ear at the time, they said, well, he can just develop speech, and it* [sign language] *isn’t necessary at all. None of that is needed. So yes, you know, you’re stepping into a new world, and you think, okay, they must know best.* (Author’s translation)

Another parent reflected on repeated experiences with professionals undermining the role of sign language:

But also, indeed, questioning the importance and necessity of sign language for ^*^child’s name^*^. […] Even recently by a speech therapist we consulted for ^*^child’s name^*^, who had actually written in her recommendation that sign language should be phased out. I said ‘Well, that’s not for you to decide. That doesn’t make sense to me at all.’ So, you really do see different views on the importance of sign language. […] Just like this itinerant practitioner who clearly has no idea either. I think, what are these people doing in their positions? I just don’t understand how you can be a professional in this field. And this person is also working with other families, telling them things like, ‘With solo equipment, you might as well… You don’t need a sign language interpreter in the classroom at all.’ (Author’s translation)

The most frequent challenges in sign language learning are presented in Table 11 below.

**Table 11:** Most commonly reported difficulties in learning NGT/NmG by parents (*N* = 20)

Code	Number of interviews mentioning code
Difficulty with language-specific components	13
Difficulty accessing courses	9
Experiencing a challenging or emotional life period	9
Limited availability of courses	9
Lack of time	8
Scarcity of courses aimed at older children or at higher-level	8
Using NGT/NmG at home or in daily life	7
Limited exposure or practice opportunities	7
Attitudes or opinions of others	5
Child’s reluctance to sign	5

*Note*. NGT = *Nederlandse Gebarentaal*; NmG = *Nederlands met Gebaren*.

Teachers also identified language-specific challenges as the most difficult for parents learning NGT, particularly in mastering NGT grammar and adjusting to its sign order. They noted that parents frequently stick to the word order of spoken Dutch, translating it directly into NGT. Additionally, two-thirds of the teachers mentioned the limited availability of study materials and the difficulties parents encountered in using NGT at home. Teachers also recognized that the language ideologies held by others, such as doctors and other professionals, posed challenges for parents. They explained that parents often received discouraging advice, with professionals often recommending focusing on spoken language or NmG instead of NGT. These professionals also frequently asserted that achieving a high level of NGT proficiency was unnecessary or unrealistic for parents. The scarcity of advanced courses, especially those designed for parents of older children, was also seen as an issue, as highlighted by one teacher:

*What I notice—and really find such a shame, but it’s largely due to money and things like that—is that parents are only able to reach a very limited level through these courses, and that very often their child quite quickly surpasses that level in terms of signing skills or usually even sooner in terms of cognitive ability or developmental stage. […] And, yes, what breaks my heart a bit as a sign language teacher is that […] after following the courses, parents actually aren’t able to read books for children aged three and a half or older, let alone true preschool books, in NGT. They simply can’t.* (Author’s translation)

Other challenges mentioned by teachers were the requirement of Dutch reading skills to follow courses and a lack of motivation among some parents. Teachers noted that parents’ motivation often differs from that of other language learners, as these parents need to learn sign language for their DHH child rather than out of intrinsic interest. One teacher explains:

*The motivation is very different. They have to learn sign language. This can sometimes be very sensitive. You have parents who are highly motivated. But you also have parents who learn it because they have to for their child. So, that drive to practice isn’t really there yet. And that often develops later, but not always in the first or second course. And that’s a challenge you encounter in class.* (Author’s translation)

An overview of the most frequently mentioned challenges for parents learning NGT, as reported by teachers, is provided in Table 12 below.

**Table 12:** Most commonly reported difficulties in learning NGT by teachers (*N* = 6)

Code	Number of interviews mentioning code
Difficulty with language-specific components	6
Using NGT at home	4
Lack of study materials	4
Acceptance of sign language or child’s hearing loss	3
Attitudes or opinions of others	3
Lack of time	2
Lack of exposure/signing environment	2
Scarcity of courses aimed at older children or at higher-level	2
Child’s reluctance to sign	2
Reading skills required	2
Getting used to a DHH teacher	2
Lack of motivation	2
Limited availability of courses	2
Experiencing a challenging or emotional life period	2

*Note*. DHH = deaf and hard-of-hearing; NGT = *Nederlandse Gebarentaal*.

When comparing parents and teachers regarding the general challenges they named, the high degree of alignment is striking. All challenges reported by parents (as shown in Table 11), except difficulty accessing courses, were also mentioned by at least two-thirds of the teachers (see Table 12). This suggests that the teachers in our sample are highly aware of the challenges parents face.

Teachers, however, identified a few additional challenges not raised by parents. These included the requirement of Dutch reading skills to follow courses and a lack of motivation, which was sometimes linked to difficulties in accepting the child’s hearing loss. A possible explanation for why these challenges were not mentioned by parents is discussed in the section *Strengths and Limitations*.

### Language options and goals

Something that stood out from the conversations with both parents and teachers was that the different language and communication options were discussed frequently. Many parents felt they needed to choose or balance between spoken Dutch, NGT, and NmG, often feeling unsure about the best option for their child. In most cases, parents and teachers did not mention this as a challenge in sign language learning per se, but as a difficult decision they had to make prior to learning sign language or as something they were still debating on. Since this was brought up frequently and often extensively, it will be discussed in this section.

Parents mentioned that they did not receive clear information about the different language and communication options or the potential impact of choosing one option over the other on their child’s language development. Many parents further mentioned receiving conflicting or negative advice about the use of sign language from professionals, making it unclear what to opt for. For example, some parents were advised against using NGT by speech therapists, observed a strong focus on speech rather than sign language from specialized organizations, or were told by specialized organizations or itinerant practitioners that their child did not need sign language.

Several parents mentioned that not knowing to what extent their child would depend on sign language in the future made the language and communication options difficult to choose from. Parents worried about their child’s spoken Dutch development if they only used sign language and therefore wanted to balance between sign and spoken language with their DHH child. One parent said that their child’s speech therapist wanted them to phase out sign language, and therefore this parent currently switched between NGT and NmG with their child while wondering if they should only use NmG in the future. Another parent noticed their child benefiting a lot from sign language but was worried that if they did not provide speech input as well, their child would not want to use speech anymore. One parent explained:

*We’ve never really had that strong drive, like, ‘We’re going to fully commit to this’. In the sense that we’d be able to sign as well as speak Dutch. I’m also still very unsure about whether we should do that* [learn sign language]*, or if that’s necessary. […] So, yeah, I still find it very difficult. In the sense that, to what extent should we pursue this? […] If it is needed, then we’d absolutely do it willingly. There are also quite a few studies now showing that children who learn a lot of sign language perform less well auditorily. And then I think, well, our entire environment is auditory. If we get really proficient at signing, then she’ll be able to communicate very well with us, but what about with her grandpa and grandma, cousins, aunts, and uncles? So, yes, part of me… I just hope she can engage in the speaking world. And if that’s not the case, then we’ll support her and guide her. But that’s something we are still struggling with. What really is best for her?* (Author’s translation)

This parent’s hesitation and concerns about sign language reflect the complex and often conflicting advice parents receive regarding the impact of sign language on their child’s language development. While some studies claim that sign language negatively affects spoken language outcomes (e.g., Geers et al., 2017), these claims have been heavily refuted by several researchers on methodological and ethical grounds (e.g., Chen Pichler, 2022; Corina & Schaefer, 2017; Hall et al., 2019; Martin et al., 2017). Moreover, researchers warn that such conclusions are harmful, given the risks of language deprivation to the cognitive and social–emotional development of DHH children (Hall et al., 2019). Recent evidence does not support the claim that sign language impedes spoken language outcomes (e.g., Chen Pichler, 2022; Humphries et al., 2024; Pontecorvo et al., 2023). On the contrary, it shows that sign language supports spoken language development and that it can protect children from the lifelong consequences of language deprivation (Chen Pichler, 2022). However, these unfounded claims have persisted over time, shaping parental anxieties and complicating decision-making around language and communication options.

Several parents struggled with balancing between spoken and sign language when both their hearing and DHH children were present or did not know which language or communication method to use at the dinner table to ensure that all children, whether hearing or DHH, could participate equally in family conversations.

Cochlear implantation was also often mentioned as something that influenced language decisions. Some parents began using NGT but, after their child was implanted, used more or only NmG. Other parents initially learned NmG because of expectations*—*often heavily influenced by specialists*—*that their child would still develop speech or become “hearing” after cochlear implantation but later switched to NGT when these expectations were not met. One parent mentioned feeling uncertain about the future of NGT, given that most children receive cochlear implants nowadays, and wondered if it would therefore be better to focus on NmG.

In one interview, a parent expressed frustration with the ongoing discussions on the different language and communication options:

*Well, the frustration is that there is so much fuss about this. Because all the time we spend repeating this could have been spent on learning sign language. Then we’d be four times as good by now.* (Author’s translation)

This parent’s frustration mirrors previous findings, which highlight how these persistent debates within clinical and educational contexts can complicate parental decision-making and hinder sign language learning (Harris et al., 2021; Humphries et al., 2024; McKee & Smiler, 2016).

Most teachers also discussed the language and communication options in their interviews and mentioned parents being pressured to not use sign language or to focus on NmG instead of NGT. They mentioned doctors telling parents that sign language would harm their child’s language development and specialized organizations informing parents that they do not have NGT materials available but only offer NmG courses. One teacher mentioned dealing with NmG materials but still offering NGT courses if parents preferred. This, however, meant they could only use the vocabulary and certain exercises from the standard course and needed to create their own materials for all other aspects. Another teacher pointed out that parents are often unaware of the differences between NGT and NmG, and that courses spend insufficient time explaining this distinction.

### Patterns and co-occurrences in the data

To explore potential patterns in the data, binary matrices were created to indicate which participants mentioned specific course positives or points for improvement, specific additional material strengths or points for improvement, and general challenges. Only items mentioned by at least a quarter of the parent participants and one-third of the teacher participants were included in this analysis, corresponding to Tables 3–12. With this analysis, we examined how often these codes appeared together, to identify potential correlations. For example, it was assessed whether participants who reported a certain general challenge were also likely to mention a specific positive or negative related to their sign language learning experiences.

Overall, few reliable correlations were found. Participants who mentioned many positives in their course experiences were also more likely to mention a greater number of negatives and general challenges. This suggests that some participants were simply more talkative or inclined to provide more extensive feedback, rather than there being a link between general challenges and course feedback.

As might be expected, a consistent pattern was that difficulty accessing courses often co-occurred with limited course availability. This suggests that, in addition to structural barriers caused by parental courses being embedded in the Dutch healthcare system, limited availability of courses in general also contributed to access challenges. This interpretation was supported by a targeted re-reading of the interview transcripts, which showed that participants who reported access challenges often also referred to limited course availability as a factor that made accessing courses more difficult.

Another notable—and perhaps unsurprising—co-occurrence was found between children’s reluctance to sign and the challenge of using NGT or NmG at home. Parents frequently reported that their child’s refusal to sign made it difficult to integrate sign language into everyday life, indicating a direct impact of the child’s attitude on home language practices.

A potentially meaningful pattern was found between the general challenge of experiencing a difficult life period and the course-related positives that most frequently co-occurred with this challenge. Among those who mentioned this challenge (*N* = 9), five referred to the opportunity for family involvement as a positive aspect of their course, five mentioned having a pleasant or skilled instructor, and four mentioned contact with other hearing parents of DHH children. While it is possible that these participants simply paid more attention to emotional factors, it may also indicate that these specific course elements were considered emotionally supportive during the difficult life period. This points to the potential of such elements—family involvement, skilled instructors, and peer connection—to help alleviate some of the emotional strain experienced by parents in the early stages of being a parent to a child with hearing loss.

## Discussion

This study investigated the experiences of Dutch parents learning Dutch Sign Language (NGT) or Sign-supported Dutch (NmG), using insights from semi-structured interviews with 21 parents and 6 NGT teachers. The findings revealed both learning experiences previously identified in the literature and unique experiences shaped by the specific parental support system in the Netherlands.

### Comparison with international contexts

In the Netherlands, the NGT teacher training program admits both hearing and DHH students, although significantly more hearing students graduate. As a result, it is very common for NGT to be taught by hearing second-language learners. The Dutch parents in this study often emphasized the value of being taught by a DHH teacher—particularly because many of the parents who mentioned this as a positive had taken courses with both DHH and hearing instructors and were therefore able to make a direct comparison. Parents in our sample valued DHH teachers not only for the language models they provided but also for their role as meaningful DHH role models who offered guidance and reassurance.

In line with international findings (e.g., Chen Pichler, 2021; Decker & Vallotton, 2016), Dutch parents and teachers felt that courses did not provide sufficient grammar instruction and courses often did not make parents self-reliant in sign language. There was a clear demand for follow-up or advanced-level courses. Teachers further emphasized the importance of using visual materials rather than text-based resources. While previous studies often linked the disadvantage of focusing on written language to parents with different home languages (Willoughby, 2009, 2015), the Dutch teachers in our study identified it as a broader issue: written Dutch as the basis for teaching NGT conflicts with the visual nature of a sign language.

Another notable element in the Dutch context is that parents in our sample highly valued having teachers come to their homes during initial sign language courses. The home environment was perceived as comfortable and allowed other family members to be involved, making this a clear advantage of the general structure of parental sign language courses in the Netherlands.

While Dutch parents valued contact with other parents during later courses, participants reported not knowing any other parents of DHH children in the early stages following the identification of their child’s hearing loss. This contributed to a sense of isolation and emotional difficulty. In contrast, in Norway, parents receive paid leave to attend week-long residential courses (Arnesen et al., 2008), which offer early opportunities for interaction with other parents as well as opportunities for language immersion and reduced time constraints. These aspects were all mentioned as challenges in the Dutch context. While travel time has been identified as an additional barrier in international research (e.g., Lieberman et al., 2024), it may have been perceived as a smaller—though still existing—obstacle in the Dutch context, given the relatively small size of the Netherlands.

A key systemic feature in the Netherlands is the integration of parental sign language courses into the healthcare system. Unlike in previous studies—where course costs and content not aimed at children were often identified as barriers to sign language learning for parents—these issues were largely absent from our Dutch sample. However, parents did face costs for additional self-study materials or courses offered outside their assigned specialized organization. Moreover, due to the way courses are tied to healthcare and funding being directed to specialized organizations rather than to parents themselves, parents were highly dependent on the offerings and communication of their assigned organization. This dependency created challenges, including large difficulty accessing courses, misalignment between course content and the child’s age due to the time of enrollment, and very restricted freedom to choose a course that best suited their wishes and family’s needs.

Both parents and teachers also expressed frustration about conflicting or discouraging advice from professionals, as has been widely documented in previous research (e.g., Dutra, 2020; Humphries et al., 2024; Mitchiner & Batamula, 2021). Like parents in earlier studies (Harris et al., 2021), Dutch parents also described wanting the best for their child but feeling uncertain about what that entailed. While earlier studies found that parents’ acceptance of their child’s hearing loss was a large challenge (Dutra, 2020; Willoughby, 2009, 2012), this theme was less prominent among Dutch parents in our study. Teachers, however, did identify acceptance as an important factor and additionally mentioned limited Dutch reading skills as a potential barrier for parents (see *Strengths and Limitations* for a possible explanation of these differences between our parent and teacher participants). Limited reading skills in the majority language, as well as the dependence of courses on that language, have also been reported in many other countries—particularly in studies focusing on parents of DHH children with a migration background (Willoughby, 2009, 2015).

Finally, many parents struggled with using sign language at home—particularly when children resisted signing in favor of spoken language. This challenge is neither unique to the Dutch context nor specific to DHH children, as it is frequently identified among children learning minority languages. These children often reject the minority language in favor of the majority language used in their broader environment (Borghetti et al., 2025; Gafaranga, 2010).

Overall, the findings from this study both reinforce and extend existing research on hearing parents’ experiences with sign language learning. While Dutch parents shared many of the same challenges reported internationally—such as inconsistent professional advice, insufficient grammar instruction, and time constraints—certain aspects of the Dutch support system, such as the predominance of hearing teachers and the integration of courses into the healthcare system, shaped their experiences in distinctive ways. These findings underscore the importance of including a wider range of national contexts in research on parental experiences with sign language learning, especially given that most existing studies have focused primarily on the United States.

### Strengths and limitations

We believe our participant group was robust for qualitative research in this context. Given the relatively small population of hearing parents of DHH children learning sign language and NGT teachers teaching parents, our sample size was comprehensive. Additionally, including participants from the southern, central, and northwestern regions of the Netherlands provided a broad overview of experiences.

Nonetheless, our participant group lacked diversity in educational level and language background. While we did not formally collect this data, our recruitment strategy—inviting parents learning NGT or NmG to participate in scientific research communicated in written Dutch—likely attracted individuals who were highly motivated, highly educated, and proficient in written Dutch. This may explain differences in the challenges reported by parents and those identified by teachers. For instance, teachers noted challenges such as parents’ lack of motivation or limited Dutch reading skills, which were not brought up by parent participants. In contrast, the parents in our sample often described the courses as too repetitive or lacking in challenge or depth. This likely reflects the high motivation levels among our participants; in a more balanced participant group, this point may have been less prominent.

It is further important to note that the population of DHH children is highly diverse. For example, data from the United States indicate that 41% of DHH students have additional disabilities (Office of Research Support and International Affairs, 2014). In the Netherlands, 12% of DHH individuals are first-generation immigrants (compared to 28% in the general Dutch population) and 20% are second-generation immigrants (compared to 10% in the general Dutch population), reflecting substantial ethnic diversity (Heppe et al., 2022). Despite this, we did not collect information on participants’ ethnic or language backgrounds or on additional disabilities among their children, nor did we intentionally balance for such diversity in our sample. As a result, the experiences identified in this study may not fully represent the broader population of parents of DHH children in the Netherlands. Previous research shows that hearing parents from ethnic minority or migration backgrounds may face additional challenges, such as incorporating an ethnic language into their family language policy (e.g., Willoughby, 2009). Limited proficiency in the majority language can further complicate access to and evaluation of information about communication options and may make it more difficult to follow sign language courses—particularly when these rely heavily on the written form of the majority language (Willoughby, 2015). This latter issue may be especially prominent in the Dutch context, where many courses focus on NmG and thus place a strong emphasis on Dutch, with parents having limited influence over whether they are enrolled in NmG or NGT courses. Given the increasingly diverse population in the Netherlands, it is important that future research explores the experiences of these different subgroups.

Regarding the teacher participants, two teachers were colleagues at the same school and used the same course materials, which likely contributed to the alignment of their responses. Collecting more demographic information from both parents and teachers would therefore have added valuable context to our results.

### Implications and recommendations

While some challenges in learning sign language—such as difficulties with language-specific features and limited practice opportunities—may not be easily resolved, others can be addressed through targeted support and systemic future improvements.

#### Supporting parents at the start of their learning trajectory

In the beginning, learning sign language feels overwhelming for many parents, especially during the challenging period following the diagnosis of their child’s hearing loss. Providing home-based lessons in a familiar and trusted environment—as is typical for initial courses in the Netherlands—can help reduce these feelings of overwhelm. Furthermore, the involvement of other family members in these courses can encourage collective learning and social support.

The inclusion of DHH teachers is also essential. They serve not only as language models but also as DHH role models who can offer reassurance and guidance to parents. In the Netherlands, however, most NGT teachers are hearing and new signers of NGT—unlike in many other countries. This is mainly due to the structure of the teacher training program, where most students are hearing. When parents do not have early exposure to DHH adults, negative ideologies about hearing loss may persist. Conversely, interacting with DHH adults in various (professional) roles can shift perspectives and thereby contribute to more positive outcomes for DHH children. Therefore, it should be part of specialized organizations’ responsibilities to involve DHH adults in early intervention, for example, through the inclusion of DHH teachers and/or itinerant practitioners. Research has already emphasized the critical role that DHH adults can play in guiding hearing parents through the social, cultural, and linguistic challenges of raising a DHH child (Chen Pichler, 2021). This aligns with international recommendations, such as those formulated by the Joint Committee on Infant Hearing in the United States (Joint Committee on Infant Hearing, 2013, p. 1338) and by Moeller et al. (2013), which emphasize that parents of DHH children should have opportunities to meet DHH adults as soon as possible after the child’s identification of hearing loss.

#### Navigating language options: the need for clear and balanced information

Although not a primary focus of the study, many parents mentioned difficulties navigating the various language and communication options (NGT, spoken Dutch, and NmG). Conflicting or incorrect advice from professionals was a common source of frustration. Professional guidance strongly influences parental decision-making (e.g., Crowe et al., 2014; Willoughby, 2015), and therefore conflicting or incomplete advice is discouraging and harmful (Humphries et al., 2024).

In the Netherlands, there has been a growing shift over the last two decades toward focusing on the speech development of DHH children, through a combination of speech therapy and the use of NmG, rather than on their sign language development (Knoors & Marschark, 2012). This shift has been heavily influenced by the advances in universal newborn hearing screening and the increasing uptake of cochlear implants, which has led scholars to claim that bilingual language policy for DHH children should be revisited (Knoors & Marschark, 2012). However, research has repeatedly emphasized the critical importance of exposing children to a fully accessible, natural language from the start—regardless of modality or majority language (e.g., Cheng et al., 2023; Hall, 2017; Hall et al., 2019; Mayberry, 2007). In the Dutch context, Hoiting and Slobin (2002) demonstrated that hearing parents who learned NGT were more capable of providing meaningful and varied sign input to their DHH children than those using NmG. They further found that children exposed to NGT produced longer utterances, more complex verbs, more questions, and greater variation in sentence types overall than children only exposed to NmG.

Even if a DHH child receives cochlear implants, the risks of relying solely on spoken language input or NmG are too great. Infants need full access to language from birth. In the Netherlands, most DHH children receive cochlear implants between 6 and 11 months of age (Bruijnzeel et al., 2017). Before that—and sometimes also after that—the only language that is accessible to them is a natural sign language. Furthermore, outcomes in spoken language development among children with cochlear implants vary widely, and it takes time for children to adapt to using the device. Even when implantation is successful, speech remains partially inaccessible, whereas sign language remains fully sensorily accessible. Moreover, sign language acquisition and comprehension are more evenly distributed among DHH children than acquisition and comprehension of sign-supported systems (Scott & Henner, 2021). While NmG may appear easier to learn than NGT for hearing parents—a claim sometimes made in the literature (Knoors & Marschark, 2012)—there is no empirical evidence to support this assumption. In fact, research shows that sign-supported systems such as NmG are cognitively demanding and prone to error: users often struggle with fluency, omit signs, or produce them inaccurately, possibly due to the processing load of simultaneous speech and signing (Akamatsu et al., 2002; Bolier, 2008). Also, the use of signed systems may overlook existing power dynamics that shape individual language choices (De Meulder et al., 2019), and their effectiveness heavily depends on who is producing or receiving (Kusters et al., 2021). In addition, NmG is based on Dutch and thus particularly difficult for parents with a non–Dutch-language background or those who speak another language at home.

Moreover, language use in DHH-hearing families is rarely binary (Arnesen et al., 2008; De Meulder et al., 2022). Communication typically shifts over time and across situations and typically includes a combination of a spoken language, a sign language, sign-supported speech, and other blended or translanguaging strategies. Professionals should refrain from pressuring parents into making categorical choices. In fact, it is harmful to do so. For DHH children growing up in the Netherlands, acquiring both NGT and Dutch (and in multilingual families, additional home languages) is critical. NGT because, among the country’s official languages, this is the only one that is fully sensorily accessible to them, in which they can freely express themselves given they receive sufficient and high-quality linguistic input, and which allows them to connect with and become part of the DHH community. And Dutch because it is the country’s majority language, used in many social, educational, and professional contexts. If parents learn a natural sign language, they can—especially with appropriate support—make more informed decisions about how to combine spoken language with supported signs. Professionals should provide complete information, emphasizing the importance of both NGT and Dutch for DHH children and actively move away from the harmful misconception that parents need to choose between the two or that combining the two simultaneously is somehow better or easier.

#### Improving course content and structure

In our study, both parents and teachers noted that many sign language courses are overly focused on vocabulary, often at the expense of sentence construction. Beyond this, essential aspects such as interaction and language development through socialization are frequently overlooked. Especially given that many parents have limited opportunities to practice signing in their daily lives, courses must incorporate interactional strategies from the start. Research by Zarchy and Geer (2023), for example, demonstrates how these elements can be effectively integrated into a sign language course.

Parents in our study also highlighted time constraints and a lack of immersion into the language. Countries like Norway provide valuable models: parents there are given work leave to attend week-long immersive sign language courses on location (Arnesen et al., 2008; Haualand & Holmström, 2019). These not only jumpstart learning but also create early opportunities to get to know other parents and DHH adults—something parents in our study highly valued.

#### Systemic and structural barriers

Unlike other countries where costs were a common barrier, participants in our study generally did not report financial obstacles to accessing sign language courses, since these courses fall under healthcare coverage. However, this system has important drawbacks as well. First, healthcare insurance only covers introductory-level courses. And second, it only covers courses that are offered through organizations that are officially recognized as healthcare providers. There are only a few such organizations in the Netherlands providing sign language courses. Each region in the country is served by one of these organizations. This means, effectively, that parents are entirely dependent on the organization that serves their region for access to courses. If the organization has a very limited offer of sign language courses—as is the case in many regions—parents have no alternative, at least not one that is covered by insurance. Similarly, if the organization only offers NmG rather than NGT courses, or only offers courses taught by a hearing L2 learner of NGT rather than a DHH L1 user of the language, parents have no choice. Most DHH NGT teachers do not work within these healthcare organizations, potentially because compensation for healthcare-covered courses is significantly lower than in educational or freelance contexts (compare salary scales under the Collective Labor Agreement for Disability Care (Vereniging Gehandicaptenzorg Nederland, 2025) and those under the Collective Labor Agreement for Primary Education (PO-Raad, 2025)). Since these DHH teachers are not formally recognized as healthcare providers, the courses they offer are not eligible for reimbursement—even if parents would prefer them. This, in our view, is an important systemic problem in the Dutch context. Moreover, the classification of sign language courses as medical care reinforces a view of deafness as something that requires medical intervention.

We recommend reclassifying sign language courses for parents and other family members of DHH children as a social service, rather than medical care. This would take away parents’ dependence on their regional healthcare organization. It will also foster a more positive perspective on sign language learning, allowing parents to appreciate it as an enriching experience rather than a necessary medical intervention. Finally, if sign language courses are classified as a social service rather than medical care, it also becomes evident that they should continue beyond the early-intervention period (currently ending at age 4).

Another systemic problem is that there has traditionally been a lack of collaboration between organizations providing sign language courses. Different organizations have developed courses independently—usually with public funding—but without sharing materials or infrastructure (e.g., for online courses). We propose that government subsidies should require collaboration and sharing of resulting materials. While this may require greater upfront investment, it would ultimately reduce duplication of effort, improve consistency across courses offered by different organizations, and ensure that all parents have access to the best materials. This would directly address the issue raised by both parents and teachers in this study regarding the frequent use of outdated course materials. The subsidy program *Deelkracht*^13^ sets a good example in this regard.

#### Long-term support and parents’ sign language fluency

Both parents and teachers reported that current sign language courses do not provide sufficient support for parents to become self-reliant in signing. Without ongoing opportunities to advance their skills, parents struggle to stay ahead of their children’s developmental needs. In addition, several parents described difficulties using sign language at home, particularly when their child showed a preference for spoken language and resisted signing. More continuous and tailored support, including the involvement of DHH itinerant practitioners, is needed to mitigate these difficulties.

Given the extensive body of research emphasizing the importance of sign language input for DHH children, parents must receive more long-term support in learning sign language. This includes access to more advanced courses as well as practical guidance tailored to the evolving developmental needs of their children.

## Conclusion

This study explored the experiences of Dutch hearing parents learning Dutch Sign Language (NGT) or Sign-supported Dutch (NmG), highlighting both widely recognized challenges and those shaped by the Dutch context. Parents valued initial home-based courses, the involvement of DHH teachers, and supplementary materials that could be used together by parent and child. However, they also faced significant challenges, including difficulties accessing courses, insufficient grammar instruction, and difficulties navigating conflicting advice from professionals. Despite parents’ strong desire to provide accessible language input to their children, many felt uncertain about the best language approach and how to sustain or improve their sign language proficiency after formal courses ended.

These findings underscore the urgent need for more accessible, longer-duration sign language courses and the development of tailored materials that align with children’s developmental stages. Additionally, consistent and informed professional advice on language and communication options is critical to reduce parents’ confusion and frustration. By addressing these challenges, hearing parents can be better supported in providing their DHH children with full access to language from an early age—an essential foundation for their overall development and social–emotional well-being.

## Footnotes

1 For example: NSDSK, Pento, Royal Dutch Auris Group, Royal Dutch Kentalis.

2 However, in the Netherlands, the costs for health care services, including sign language courses covered by insurance, are first deducted from the annual *eigen risico* (mandatory deductible), which parents must pay out of pocket before insurance coverage begins.

3 For instance, see Taalbeleid binnen de gezinsbegeleiding voor kinderen met een auditieve beperking.

4 But see Napier et al. (2007) for Australian Sign Language.

5 This excludes three teachers who were contractually required to participate in scientific research.

6 See for example Roy (1992) on interpreter influences.

7 This initial discomfort aligns with what Sheridan (2018) describes as *exposure insecurity* in L2 sign language learners in general: the feeling of being observed and exposed due to the physicality of signing.

8 Several language learning methods make use of this “reversed order” of homework and in-class instruction, see for example Flipping the Classroom by Brame (2019).

9 Online Gebarenwoordenboek (https://ow.gebarencentrum.nl/).

10 Most popular apps and online platforms:
*Gebarenles met Lotte & Max* (https://gebarenles.nl/)
*De Kindergebarenapp* (https://www.nsdsk.nl/kindergebarenapp)
*Het gebaar van de dag* (https://www.hetgebaarvandedag.nl/)

11 Corpus NGT (https://www.corpusngt.nl/).

12 Itinerant practitioners (referred to as *ambulant begeleiders* in Dutch) support children and their parents in the home environment, offering information and guidance on a range of topics, including hearing technology, communication strategies, and language development.

13 Deelkracht (https://www.deelkracht.nl/en/).

## Appendix A—Interview Questions

*Interview questions for parents*

1. Can you describe the sign language courses you have followed?
2. What were the positive aspects of these courses?
3. Are there things you missed in these courses or other areas for improvement?
4. How could other materials fill this experienced gap?
5. Besides these courses, have you used any other learning materials? Which ones?
6. In what ways were these extra materials helpful?
7. What were the limitations of these materials?
8. What general challenges have you experienced while learning NGT/NmG?

*Interview questions for teachers*

1. Can you describe the NGT parental courses you currently teach or have taught in the past?
2. What are the positive aspects of these courses?
3. Are there things you miss in these courses or other areas for improvement?
4. How could other materials fill this experienced gap?
5. Do you use additional (self-made or already existing) materials during your lessons? Which ones?
6. In what ways are these additional materials helpful?
7. What are the limitations of these materials?
8. What are the main challenges parents experience when learning NGT?
